# Socioeconomic determinants of leprosy new case detection in the 100 Million Brazilian Cohort: a population-based linkage study

**DOI:** 10.1016/S2214-109X(19)30260-8

**Published:** 2019-07-19

**Authors:** Joilda Silva Nery, Anna Ramond, Julia Moreira Pescarini, André Alves, Agostino Strina, Maria Yury Ichihara, Maria Lucia Fernandes Penna, Liam Smeeth, Laura C Rodrigues, Mauricio L Barreto, Elizabeth B Brickley, Gerson Oliveira Penna

**Affiliations:** a Instituto de Saúde Coletiva, Universidade Federal da Bahia, Salvador, Brazil; b Department of Infectious Disease Epidemiology, London School of Hygiene & Tropical Medicine, London, UK; c Centro de Integração de Dados e Conhecimentos para Saúde, Fundação Oswaldo Cruz, Salvador, Brazil; d Epidemiology and Biostatistics Department, Federal University Fluminense, Rio de Janeiro, Brazil; e Tropical Medicine Centre, University of Brasília, Fiocruz School of Goverment Brasília, Brazil

## Abstract

**Background:**

Although leprosy is recognised as a disease of poverty, there is little evidence on the specific socioeconomic factors associated with disease risk. To inform targeted strategies for disease elimination, we investigated socioeconomic markers of leprosy risk in Brazil.

**Methods:**

Socioeconomic data from the 100 Million Brazilian Cohort were linked to the Brazilian national disease registry (Sistema de Informação de Agravos de Notificação) for leprosy from Jan 1, 2007, to Dec 31, 2014. Using Poisson regression, we assessed the association of socioeconomic factors with risk of incident leprosy in the full cohort and in children (aged 0–15 years), by leprosy subtype and region of residence.

**Findings:**

In an analysis of 23 899 942 individuals including 18 518 patients with leprosy, increased levels of deprivation were associated with an increased risk of leprosy in Brazil. Directions of effect were consistent in children younger than 15 years and across disease subtypes. Individuals residing in regions with the highest poverty in the country (central-west, north, and northeast regions) had a risk of leprosy incidence five-to-eight times greater than did other individuals. Decreased levels of income and education and factors reflecting unfavourable living conditions were associated with an up to two-times increase in leprosy incidence (incidence rate ratio 1·46, 95% CI 1·32–1·62, for lowest *vs* highest quartile of income per capita; 2·09, 95% CI 1·62–2·72, for lowest *vs* highest level of education).

**Interpretation:**

Within the poorest half of the Brazilian population, the most deprived individuals have the greatest risk of leprosy. Strategies focusing on early detection and treatment in the poorest populations could contribute substantially to global disease control.

**Funding:**

Medical Research Council, Wellcome Trust, Coordenação de Aperfeiçoamento de Pessoal de Nível Superior (Brazil), the Conselho Nacional das Fundações Estaduais de Amparo à Pesquisa, Economic and Social Research Council, Biotechnology and Biological Sciences Research Council, Conselho Nacional de Desenvolvimento Científico e Tecnológico, and Fundação de Apoio à Pesquisa do Distrito Federal.

## Introduction

Leprosy, also known as Hansen's disease, is a chronic infectious disease of the peripheral nervous system, skin, eyes, and upper respiratory tract. Although caused by a non-lethal and curable mycobacterial infection, late diagnosis and treatment of leprosy cases can result in permanent disabilities associated with social stigma and economic hardship.^1^, ^2^ Following introduction of multidrug therapy in 1981, global rates of detection of new cases of leprosy declined, but subsequently stabilised, with approximately 200 000 new cases recorded annually since 2005.^3^, ^4^ Efforts to interrupt disease transmission, which are the focus of the WHO Global Leprosy Strategy 2016–2020,^5^ are hindered by the pathogen's long incubation period (2–12 years) and gaps in knowledge regarding individual susceptibility to infection and disease development.^1^

Although leprosy is commonly recognised as a disease of poverty predominately affecting vulnerable and marginalised populations, its specific social determinants remain inadequately evaluated.^6^ As reported by Pescarini and colleagues in a 2018 systematic review,^7^ most investigations into the association between social conditions and leprosy in high-burden countries have used ecological study designs. This review^7^ also highlighted the insufficient longitudinal evidence on socioeconomic determinants of leprosy incidence.^8^, ^9^, ^10^, ^11^, ^12^, ^13^, ^14^ To achieve the goal of halting leprosy transmission, a better understanding of the risk markers of leprosy incidence will be crucial for informing targeted strategies for early case detection and treatment in high-risk populations.

The current study aims to meet this need by investigating the associations between social indicators and new leprosy case detection in Brazil, a country with active transmission and the second highest burden of cases worldwide.^15^ Using data collected routinely between 2007 and 2014 and linked as part of the 100 Million Brazilian Cohort, this study used a hierarchical framework to identify geographical, socioeconomic, and household factors associated with the new case detection rate of leprosy in the overall population, in individuals younger than 15 years, of paucibacillary and multibacillary subtypes, and in five regions of Brazil.

**Research in context**

**Evidence before this study**

The existing evidence on the social determinants of leprosy was recently summarised in a 2018 systematic review and meta-analysis. The review identified 39 studies reporting on the association of socioeconomic determinants with prevalence or incidence of leprosy across eight high-burden leprosy countries (Brazil, India, Bangladesh, Indonesia, Myanmar, Egypt, the Philippines, and Sri Lanka). Findings from 13 ecological and 27 individual-level studies (including seven prospective studies) highlighted an increased risk of leprosy with increased levels of poverty. Socioeconomic factors associated with increased leprosy risk after meta-analysis included household exposure to leprosy cases, crowded living conditions, manual labour, and food insecurity. Male sex was also associated with an increased risk of leprosy. Additional factors, which were not included in the meta-analyses but were associated with increased risk of leprosy in multiple studies, were increasing age, lack of appropriate sanitary conditions, and low levels of education. This review also highlighted the small numbers of leprosy cases available in most studies and the fact that other potentially relevant sociodemographic factors, such as race or ethnicity, were not assessed.

**Added value of this study**

We have performed the largest study of socioeconomic determinants of leprosy risk in over 33 million Brazilian individuals, which includes over 23 000 cases. This study both confirms previous findings and provides a more robust assessment of the contribution of deprivation to leprosy risk than has previously been possible. In particular, our results highlight a striking gradient of increasing leprosy risk with decreasing levels of education and income. Furthermore, this study shows evidence of large inequalities in leprosy risk across different ethnic groups in Brazil.

**Implications of all the available evidence**

Indicators of deprivation and low socioeconomic status are associated with an increased risk of leprosy in Brazil. Strategies targeting high-risk populations to reduce leprosy transmission and prevent progression towards potentially stigmatising disabilities should prioritise individuals and families living in precarious situations with low income. Social development appears to be a key strategy for reducing the prevalence of leprosy worldwide.

## Methods

### Study design and participants

The 100 Million Brazilian Cohort^16^ was created by the Centro de Integração de Dados e Conhecimentos para Saúde at Oswaldo Cruz Foundation (Salvador, Brazil) to evaluate the impact of social determinants and policies on health. The cohort includes administrative records from over 114 million individuals aged 16 years or older whose families applied for social assistance via the Cadastro Único para Programas Sociais (CadÚnico) registry between 2001 and 2015. In January, 2018, the baseline cohort was linked to leprosy records from 2007 to 2014 registered in the national notifiable disease system, Sistema de Informação de Agravos de Notificação (SINAN-leprosy).^17^, ^18^

In our analysis, the study population included members of the 100 Million Brazilian Cohort followed from Jan 1, 2007, to Dec 31, 2014. Cohort members were excluded if they were diagnosed with leprosy before registration in CadÚnico, belonged to family units without one member aged 15 years or older (ie, children registered separately from their families), had less than 1 day of follow-up, or were a recurrent leprosy case. Within each family unit, the oldest member was designated as head of family.

This study complied with the international (Helsinki), Brazilian, and UK research regulations and was approved by the ethics committees of the University of Brasília (Brasília, Brazil; reference number 1.822.125), Instituto Gonçalo Moniz-Fiocruz (Salvador, Brazil; reference number 1.612.302), and London School of Hygiene & Tropical Medicine (London, UK; reference number 10580-1).

### Procedure

For this investigation, we extracted a de-identified dataset including geographical location (region and urbanisation), exposure data related to family living conditions (water supply, household density, housing construction material, presence of electricity, and sewage and waste collection in the household), family income, and sociodemographic indicators (sex, age, race or ethnicity, education, and work) for all family members. Outcome data obtained from linkage to SINAN-leprosy included information on leprosy cases (date of diagnosis, case type [newly detected or relapsed], and leprosy type [paucibacillary or multibacillary]).

### Statistical analysis

**Figure 1:**
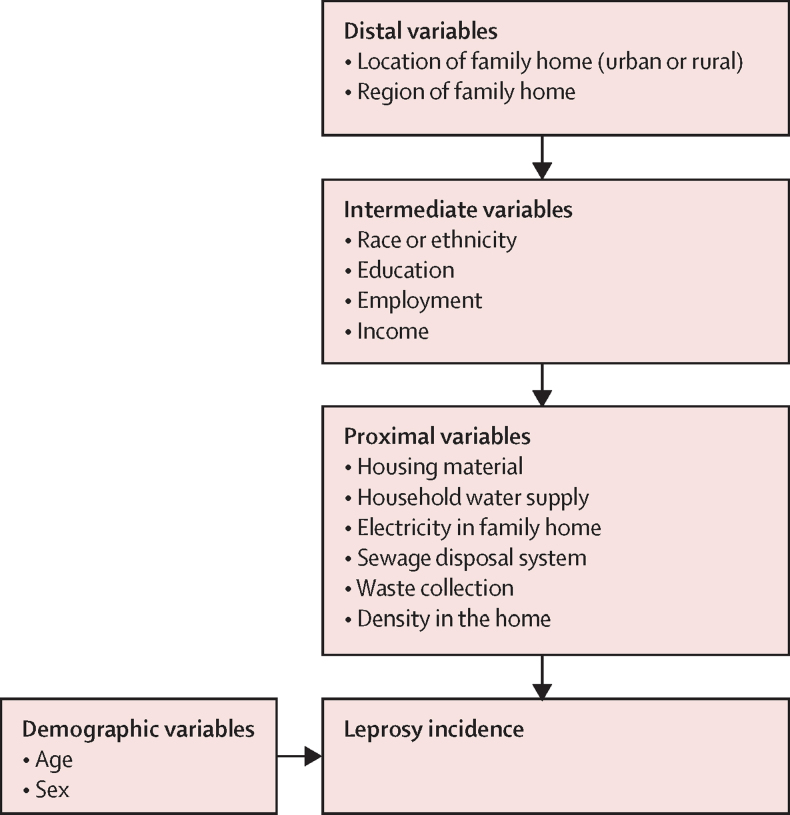
Conceptual framework for the association of sociodemographic factors with leprosy incidence

Based on a broad literature review, we developed a hierarchical framework to investigate the social determinants of leprosy in Brazil (figure 1). Social indicators were grouped into three blocks representing distal, intermediate, and proximal variables. Distal variables related to geographical factors and included region and location of the family home (urban or rural). Intermediate variables related to socioeconomic position in the community and included self-identified race or ethnicity (“preto” [ie, black], “pardo” (ie, mixed race), “branco” [ie, white], Asian, or Indigenous), education, employment, and per-capita family income (relative to the Brazilian minimum wage). For individuals younger than 18 years, education and employment were reported as the education level and employment status of the head of family. Proximal variables related to household conditions at the family level and included building material, water supply, sanitation, electricity, waste disposal destination, and household density (ie, number of individuals per room). Age and sex were considered to be confounders a priori. The primary outcome of this study was incidence of diagnosed and registered leprosy cases, commonly defined as the leprosy new case detection rate.

For each potential risk marker of leprosy, we first estimated the crude incidence of leprosy in all individuals with available data. Thereafter, we did a complete case analysis, excluding individuals with missing data on any of the covariates in the hierarchical framework.

For the primary outcome analysis, we used Poisson regression models with cluster-robust SEs (ie, accounting for familial clustering of covariates) to estimate the adjusted incidence rate ratios (IRRs) of leprosy. Following a hierarchical analytical approach, we created three adjusted models of leprosy incidence. All variables associated with IRR of leprosy at a significance threshold of p value less than 0·10 were included in the next level model. Model 1 included distal factors plus age and sex. Model 2 included variables from model 1 and intermediate factors. Model 3 included variables from model 2 and proximal factors.

In subsidiary analyses, we investigated the association of potential risk markers with leprosy incidence in the subgroup of children younger than 15 years and by clinical subtype (ie, paucibacillary *vs* multibacillary) following the same hierarchical approach as the main analysis. Follow-up was censored at the time individuals turned 15 years of age or at the time of leprosy diagnosis, whichever occurred first. We selected the younger-than-15-years age group as new case diagnosis in children is indicative of active disease transmission and this age group represents a priority target group for leprosy elimination.^5^, ^19^ Similarly, multibacillary leprosy presentations are associated with increased rates of onward transmission and disabilities than paucibacillary cases. Given the important regional inequality in socio-economic and demographic indicators in Brazil, we also investigated potential regional differences in socioeconomic determinants of leprosy in analyses stratified by region (south, southeast, northeast, north, and central-west) using the described hierarchical approach.^20^

In the sensitivity analysis, we adjusted for year of entry into the cohort to assess the potential effect of policy changes during the study period (Jan 1, 2007, to Dec 31, 2014). Further, as leprosy has a long incubation period and some baseline factors could have changed over time (eg, income or household characteristics), we did a sensitivity analysis restricted to 2 years of follow-up.

All p values were calculated for two-sided statistical tests, and all analyses were done using Stata, version 15.0.

### Role of the funding source

The funder of the study played no role in study design, data collection, data analysis, data interpretation, or writing of the report. The joint first authors had full access to all the data in the study and all authors had final responsibility for the decision to submit for publication.

## Results

**Figure 2:**
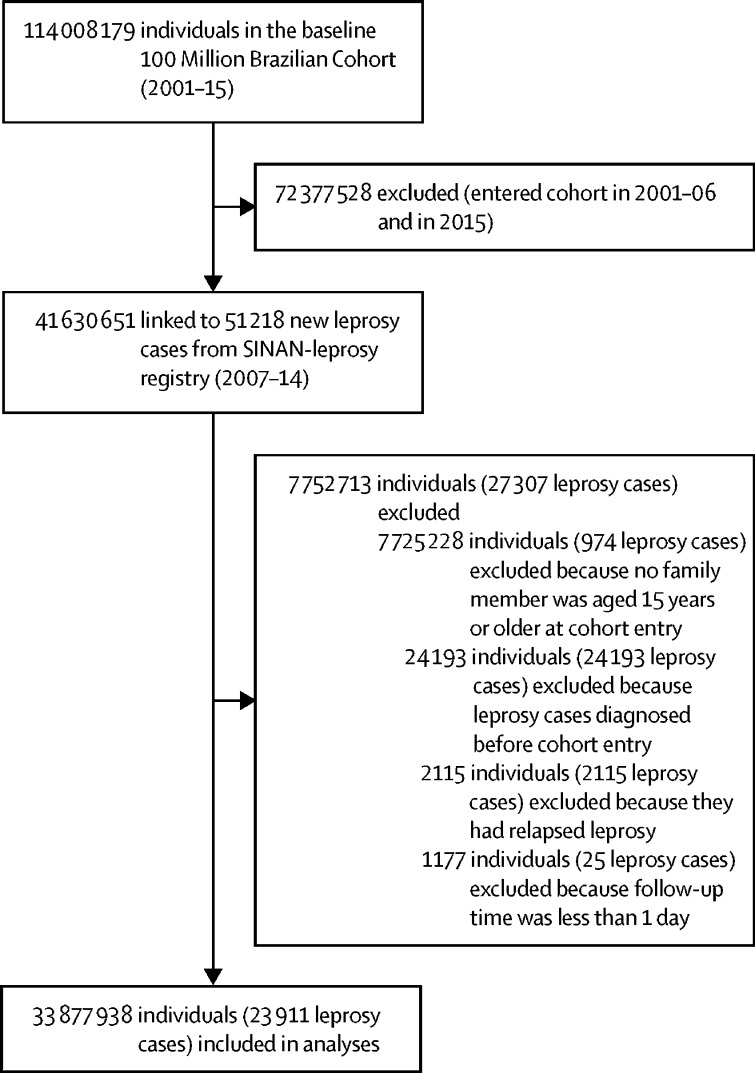
Study profile The population in this study was selected from the 100 Million Brazilian Cohort.

The study population included 33 877 938 individuals (55% female), and 23 911 incident leprosy cases (figure 2). Mean age at entry into the cohort was 24·4 years (SD 21·1) and median follow-up duration was 4·1 years (IQR 2·1–6·4), with a total of 139 778 058 person-years of follow-up. Most individuals (27 430 499 [81·0%]) lived in an urban setting, and the southeast (with 12 760 974 [37·7%] individuals) and northeast (with 10 323 799 [30·5%] individuals) regions of Brazil were the most represented in the cohort (table 1). 19 275 827 (56·9%) individuals self-identified as being “pardo” (ie, mixed race), and 11 154 507 (32·9%) identified as being “branco” (ie, white). 11 958 020 (35·3%) individuals completed education at primary school level or less, and 27 938 913 (82·5%) had a per-capita income of less than half the minimum wage.

**Table 1:** Baseline characteristics (n=33 877 938)

		Number of individuals with missing data*	**Number of individuals**	**Person-years**	**Leprosy cases**	**Crude incidence**†**(95% CI)**
**Demographic variables**						
Age		2816 (<0·1%)	..	..	..	..
	<15 years	..	14 172 096 (41·8%)	62 898 954	3273	5·2 (5·0–5·4)
	≥15 years	..	19 701 096 (58·2%)	76 867 919	20 636	26·8 (26·5–27·2)
Sex		0	..	..	..	..
	Female	..	18 499 333 (54·6%)	75 642 536	11 778	15·6 (15·3–15·9)
	Male	..	15 378 605 (45·4%)	64 137 271	12 133	18·9 (18·6–19·3)
**Distal variables**						
Region of family home		0	..	..	..	..
	South	..	3 729 784 (11·0%)	13 633 178	663	4·9 (4·5–5·2)
	Southeast	..	12 760 974 (37·7%)	48 906 390	3268	6·7 (6·5–6·9)
	North	..	4 179 048 (12·3%)	18 314 832	5525	30·2 (29·4–31)
	Northeast	..	10 323 799 (30·5%)	48 101 419	10 017	20·8 (20·4–21·2)
	Central-west	..	2 884 333 (8·5%)	10 823 988	4 438	41 (39·8–42·2)
Location of family home		36 956 (0·1%)	..	..	..	..
	Urban	..	27 430 499 (81·0%)	109 800 000	18 953	17·3 (17·0–17·5)
	Rural	..	6 410 472 (18·9%)	29 895 545	4936	16·5 (16·1–17)
**Intermediate variables**						
Race or ethnicity		943 260 (2·8%)	..	..	..	..
	“Pardo” (ie, mixed race)	..	19 275 827 (56·9%)	83 774 268	16 958	20·2 (19·9–20·6)
	“Branco” (ie, white)	..	11 154 507 (32·9%)	43 225 070	4448	10·3 (10·0–10·6)
	“Preto” (ie, black)	..	2 115 391 (6·2%)	9 241 334	1892	20·5 (19·6–41·4)
	Indigenous	..	247 685 (0·7%)	1 115 229	80	7·2 (5·8–8·9)
	Asian	..	141 268 (0·4%)	426 151	78	18·3 (14·7–22·9)
Highest level of education‡		4 027 882 (11·9%)	..	..	..	..
	Higher education	..	193 732 (0·6%)	785 443	69	8·8 (6·9–11·1)
	Year 10–12	..	7 906 287 (23·3%)	22 117 454	2338	10·6 (10·2–11·0)
	Year 6–9	..	9 792 017 (28·9%)	41 842 035	5945	14·2 (13·9–14·6)
	Year 1–5	..	8 933 482 (26·4%)	41 040 299	8839	21·5 (21·1–22·0)
	Preschool, no education, or illiterate	..	3 024 538 (8·9%)	14 461 430	4154	28·7 (24·6–29·6)
Employment‡		4 768 980 (14·1%)	..	..	..	..
	Currently employed	..	17 700 913 (52·2%)	61 157 120	10 478	17·1 (16·8–17·5)
	Unemployed student	..	4 327 199 (12·8%)	26 942 843	5335	19·8 (19·3–20·3)
	Unemployed (not student)	..	10 080 846 (29·8%)	28 555 481	5 483	19·2 (18·7–19·7)
Income per capita§		929 (<0·1%)	..	..	..	..
	>1 minimum wage	..	1 424 157 (4·2%)	3 801 664	857	22·1 (20·7–23·6)
	>0·5–1 minimum wage	..	4 513 939 (13·3%)	13 027 761	3535	27·1 (26·3–28)
	>0·25–0·5 minimum wage	..	6 438 130 (19·0%)	23 592 423	3845	16·3 (15·8–16·8)
	0–0·25 minimum wage	..	16 662 023 (49·2%)	80 578 218	12 986	16·1 (15·8–16·4)
	No income	..	4 838 760 (14·3%)	18 697 865	2687	14·4 (13·8–14·9)
**Proximal variables**						
Housing material		849 112 (2·5%)	..	..	..	..
	Brick or cement	..	26 645 109 (78·7%)	106 900 000	16 339	15·3 (15·1–15·5)
	Taipa, wood, or other	..	6 383 717 (18·8%)	31 139 685	7254	23·3 (22·8–23·8)
Household water supply		849 109 (2·5%)	..	..	..	..
	Public network	..	24 975 375 (73·7%)	100 100 000	16 110	16·1 (15·8–16·3)
	Well, natural source, cistern, or other	..	8 053 454 (23·8%)	37 981 211	7483	19·7 (19·3–20·2)
Sewage disposal system		1 577 939 (4·7%)	..	..	..	..
	Public network	..	17 084 378 (50·4%)	66 948 282	7609	11·4 (11·1–11·6)
	Septic tank, ditch, or other	..	15 215 621 (44·9%)	69 532 912	15 597	19·7 (19·3–20·2)
Electricity in family home		849 060 (2·5%)	..	..	..	..
	Home meter	..	26 683 824 (78·8%)	109 500 000	18 922	17·3 (17–17·5)
	Community meter	..	2 108 775 (6·2%)	8 110 157	838	10·3 (9·7–11·1)
	Illegal electricity, gas lighting, candlelight, or other	..	4 236 279 (12·5%)	20 483 095	3833	18·7 (18·1–19·3)
	Waste collection	849 136 (2·5%)	..	..	..	..
	Public collection system	..	26 872 031 (79·3%)	107 600 000	17 783	16·5 (16·3–16·8)
	Burned, buried, outdoor disposal, or other	..	6 156 771 (18·2%)	30 467 394	5811	19·1 (18·6–19·6)
Density (individuals per room)		865 411 (2·6%)	..	..	..	..
	≤0·5	..	9 989 012 (29·5%)	35 201 492	8687	24·7 (24·2–25·2)
	>0·5–0·75	..	7 103 940 (21·0%)	27 826 309	4296	15·4 (15–15·9)
	>0·75–1·00	..	7 806 338 (23·0%)	33 761 337	5007	14·8 (14·4–15·3)
	>1·00	..	8 113 237 (23·9%)	41 223 277	5583	13·5 (13·2–19·9)

Data are n or n (%) unless otherwise specified.

* Individuals with missing data were included in the study population and for the estimation of crude incidence, but not in the adjusted analysis (table 2).

† The crude incidence is expressed per 100 000 person-years.

‡ Information on education and employment is reported for adult individuals (>18 years) or for the oldest member of the family of individuals younger than 18 years.

§ Income in minimum wage was calculated by year, dividing the familial income by the minimum wage in the year of application in the Cadastro Único para Programas Sociais.

The association of socioeconomic risk factors with incidence of leprosy was assessed in a complete case analysis of 23 899 942 individuals, including 18 518 leprosy cases, followed up for a median of 3·5 years (IQR 2·0–6·6; table 2). Subgroup analyses in children younger than 15 years included 9 772 650 children and 2725 leprosy cases (table 3). Geographical factors included in the distal model were associated with leprosy risk in the full cohort and in children. In the full cohort, individuals residing in the north or central-west regions had an approximately eight-times higher leprosy incidence than did individuals in the south region (IRR 8·01, 95% CI 7·29–8·80 [north *vs* south regions], and 8·36, 7·60–9·20 [central-west *vs* south regions]; table 2). This risk was enhanced in individuals younger than 15 years; children living in the north region had a 34-times higher leprosy incidence than did those living in the south region (33·6, 18·1–62·2; table 3). Individuals living in an urban area were also at increased risk of leprosy compared with rural inhabitants (1·22, 1·18–1·27, in the full cohort; 1·45, 1·29–1·63, in children).

**Table 2:** Multivariate hierarchical association of socioeconomic factors with leprosy incidence using a complete-case analysis (n=23 899 942)

		**Model 1**		Model 2*		**Model 3**†	
		IRR	p value	IRR	p value	IRR	p value
**Demographic variables**							
Age (per 10 years)		1·31 (1·30–1·31)	<0·001	1·35 (1·34–1·36)	<0·001	1·36 (1·35–1·36)	<0·001
Sex							
	Female	Ref		..	..	..	..
	Male	1·25 (1·22–1·29)	<0·001	1·23 (1·19–1·26)	<0·001	1·22 (1·19–1·26)	<0·001
**Distal variables**							
Region of family home							
	South	Ref		..	..	..	..
	South-east	1·47 (1·33–1·61)	<0·001	..	..	..	..
	North-east	5·09 (4·64–5·57)	<0·001	..	..	..	..
	North	8·01 (7·29–8·80)	<0·001	..	..	..	..
	Central-west	8·36 (7·60–9·20)	<0·001	..	..	..	..
Location of family home							
	Rural	Ref		..	..	..	..
	Urban	1·22 (1·18–1·27)	<0·001	..	..	..	..
**Intermediate variables**							
Race or ethnicity							
	“Branco” (ie, white)	..	..	Ref		..	..
	“Preto” (ie, black)	..	..	1·40 (1·32–1·49)	<0·001	..	..
	“Pardo” (ie, mixed race)	..	..	1·26 (1·21–1·32)	<0·001	..	..
	Asian	..	..	1·12 (0·87–1·45)	0·38	..	..
	Indigenous	..	..	0·39 (0·30–0·51)	<0·001	..	..
Highest level of education‡							
	Higher education	..	..	Ref		..	..
	Year 10–12	..	..	1·39 (1·07–1·80)	0·01	..	..
	Year 6–9	..	..	1·77 (1·37–2·29)	<0·001	..	..
	Year 1–5	..	..	1·95 (1·51–2·52)	<0·001	..	..
	Preschool, no education, or illiterate	..	..	2·09 (1·62–2·72)	<0·001	..	..
Employment‡							
	Currently employed	..	..	Ref		..	..
	Unemployed student	..	..	0·96 (0·92–0·99)	<0·001	..	..
	Unemployed (not student)	..	..	0·73 (0·69–0·76)	<0·001	..	..
Income per capita							
	>1 minimum wage	..	..	Ref		..	..
	>0·5–1 minimum wage	..	..	0·95 (0·87–1·05)	0·31	..	..
	>0·25–0·5 minimum wage	..	..	1·23 (1·12–1·35)	<0·001	..	..
	0–0·25 minimum wage	..	..	1·47 (1·34–1·61)	<0·001	..	..
	No income	..	..	1·46 (1·32–1·62)	<0·001	..	..
**Proximal variables**							
Housing material							
	Brick or cement	..	..	..	..	Ref	
	Taipa, wood, or other	..	..	..	..	1·34 (1·29–1·40)	<0·001
Household water supply							
	Public network	..	..	..	..	Ref	
	Well, natural source, cistern, or other	..	..	..	..	0·97 (0·93–1·01)	0·16
Sewage disposal system							
	Public network	..	..	..	..	Ref	
	Septic tank, ditch, or other	..	..	..	..	1·35 (1·30–1·40)	<0·001
Electricity in family home							
	Home meter	..	..	..	..	Ref	
	Community meter	..	..	..	..	0·93 (0·86–1·01)	0·10
	Illegal electricity, gas lighting, candlelight, or other	..	..	..	..	1·03 (0·98–1·08)	0·26
Waste collection system							
	Public collection system	..	..	..	..	Ref	
	Burned, buried, outdoor disposal, or other	..	..	..	..	0·97 (0·92–1·04)	0·41
Density (individuals per room)							
	≤0·5	..	..	..	..	Ref	
	>0·5–0·75	..	..	..	..	1·02 (0·97–1·07)	0·37
	>0·75–1·00	..	..	..	..	1·01 (0·97–1·06)	0·61
	>1·00	..	..	..	..	0·97 (0·92–1·02)	0·25

IRRs for leprosy new case detection were obtained using generalised linear Poisson models with clustered SEs to account for clustering by family. A complete-case analysis approach was used excluding from all models individuals with missing data in any of the three models. IRR=incidence rate ratio.

* Covariates in model 2 were adjusted for covariates from model 1 with p<0·1 (ie, model 2 was adjusted for region and location of family home).

† Covariates in model 3 are adjusted for covariates from model 1 and model 2 with p<0·1 (ie, model 3 was adjusted for region, location of family home, ethnicity, education, employment, and income per capita).

‡ Information on education and employment is reported for adult individuals (>18 years) or for the oldest member of the family of individuals younger than 18 years.

**Table 3:** Multivariate hierarchical association of socioeconomic factors with leprosy new case detection in individuals younger than 15 years (n=9 772 650)

		**Model 1**		Model 2*		**Model 3**†	
		IRR	p value	IRR	p value	IRR	p value
**Demographic variables**							
Age (years)							
	0 to <5	Ref	..	Ref	..	Ref	..
	≥5 to <10	2·82 (2·55–3·12)	<0·001	2·70 (2·44–2·98)	<0·001	2·71 (2·45–3·00)	<0·001
	≥10 to <15	3·34 (2·94–3·80)	<0·001	3·28 (2·89–3·73)	<0·001	3·33 (2·92–3·80)	<0·001
Sex							
	Female	Ref	..	Ref	..	Ref	..
	Male	0·98 (0·90–1·07)	0·61	0·98 (0·90–1·06)	0·57	0·98 (0·89–1·06)	0·58
**Distal variables**							
Region of family home							
	South	Ref	..	..	..	..	..
	Southeast	5·44 (2·91–10·16)	<0·001	..	..	..	..
	Northeast	24·18 (13·08–44·74)	<0·001	..	..	..	..
	North	33·58 (18·12–62·24)	<0·001	..	..	..	..
	Central-west	26·02 (13·94–48·56)	<0·001	..	..	..	..
Location of family home							
	Rural	Ref	..	..	..	..	..
	Urban	1·45 (1·29–1·63)	<0·001	..	..	..	..
**Intermediate variables**							
Race or ethnicity							
	“Branco” (ie, white)	..	..	Ref	..	..	..
	“Preto” (ie, black)	..	..	1·92 (1·52–2·42)	<0·001	..	..
	“Pardo” (ie, mixed race)	..	..	1·60 (1·38–1·85)	<0·001	..	..
	Asian	..	..	1·92 (0·91–4·07)	0·09	..	..
	Indigenous	..	..	0·35 (0·17–0·75)	0·007	..	..
Highest level of education (head of family)							
	Higher education	..	..	Ref	..	..	..
	Year 10–12	..	..	1·49 (0·61–3·62)	0·38	..	..
	Year 6–9	..	..	2·12 (0·88–5·10)	0·10	..	..
	Year 1–5	..	..	2·14 (0·89–5·17)	0·09	..	..
	Preschool or no education or illiterate	..	..	2·66 (1·10–6·49)	0·03	..	..
Employment (head of family)							
	Currently employed	..	..	Ref	..	..	..
	Unemployed student	..	..	1·08 (0·96–1·21)	0·38	..	..
	Unemployed (not student)	..	..	0·70 (0·59–0·82)	<0·001	..	..
Income per capita							
	>1 minimum wage	..	..	Ref	..	..	..
	>0·5–1 minimum wage	..	..	2·61 (0·35–19·17)	0·35	..	..
	>0·25–0·5 minimum wage	..	..	3·44 (0·48–24·56)	0·22	..	..
	0–0·25 minimum wage	..	..	4·31 (0·61–30·58)	0·14	..	..
	No income	..	..	4·01 (0·56–28·60)	0·17	..	..
**Proximal variables**							
Housing material							
	Brick or cement	..	..	..	..	Ref	..
	Taipa, wood, or other	..	..	..	..	1·26 (1·11–1·43)	<0·001
Household water supply							
	Public network	..	..	..	..	Ref	
	Well, natural source, cistern, or other	..	..	..	..	0·95 (0·84–1·08)	0·44
Sewage disposal system							
	Public network	..	..	..	..	Ref	..
	Septic tank, ditch, or other	..	..	..	..	1·55 (1·37–1·75)	<0·001
Electricity in family home							
	Home meter	..	..	..	..	Ref	..
	Community meter	..	..	..	..	0·90 (0·71–1·15)	0·41
	Illegal electricity, gas lighting, candlelight, or other	..	..	..	..	1·19 (1·05–1·35)	0·01
Waste collection system							
	Public collection system	..	..	..	..	Ref	..
	Burned, buried, outdoor disposal, or other	..	..	..	..	0·79 (0·68–0·93)	0·004
Density (individuals per room)							
	≤0·5	..	..	..	..	Ref	..
	>0·5–0·75	..	..	..	..	1·22 (1·01–1·46)	0·04
	>0·75–1·00	..	..	..	..	1·15 (0·96–1·38)	0·22
	>1·00	..	..	..	..	1·21 (1·01–1·44)	0·04

IRRs for leprosy new case detection were obtained using generalised linear Poisson models with clustered SEs to account for clustering by family. A complete-case analysis approach was used excluding from all models individuals with missing data in any of the three models. Follow-up time was censored when individuals turned 15 or were diagnosed with leprosy, whichever event occurred first. IRR=incidence rate ratio.

* Covariates in model 2 were adjusted for covariates from model 1 with p<0·1 (ie, model 2 was adjusted for region and location of family home).

† Covariates in model 3 are adjusted for covariates from model 1 and model 2 with p<0·1 (ie, model 3 was adjusted for region, location of family home, ethnicity, education, and employment; model 3 was also adjusted for income per capita despite p>0·1 because it was considered a relevant confounder).

Intermediate factors associated with risk of leprosy in the full cohort included race or ethnicity, education, and income (table 2). When compared with individuals in the full cohort identifying as “branco” (ie, white), those who identified as “preto” (ie, black) had the highest risk of leprosy (IRR 1·40, 95% CI 1·32–1·49), followed by “pardo” individuals (1·26, 1·21–1·32). Indigenous individuals appeared to have lower leprosy incidence than did the “branco” population (0·39, 0·30–0·51). Lower education level and low income were also associated with increased leprosy risk, with evidence of a positive trend for both factors. The greatest increase in risk was found for individuals with the lowest level of education (or whose head of family had the lowest level of education), with an over two-times higher leprosy incidence than those who continued education past high school (2·09, 1·62–2·72). Similarly, individuals with no income or an income per capita less than 0·25 times the minimum wage had a leprosy risk more than 40% higher than those earning more than minimum wage (1·46, 1·32–1·62, for individuals with no income; 1·47, 1·34–1·61, for those with incomes <0·25 times the minimum wage). Unemployment was associated with a reduced leprosy risk regardless of current education status. In further analyses investigating this inverse effect, we identified an interaction between age and employment after stratification by year of cohort entry (as the employment questionnaire was updated in 2010; appendix p 1). After stratification, only the 18–30-years age group showed a significant protective effect of unemployment in both subgroups.

The association of self-reported race or ethnicity with leprosy risk in children followed a similar pattern to the full cohort, with the highest risk among children identifying as “preto” (IRR 1·92, 95% CI 1·52–2·42), followed by those identifying as “pardo” (1·60, 1·38–1·85), and a decreased risk for Indigenous children (0·35, 0·17–0·75). Having a head of family in the lowest education group was also associated with a significantly increased leprosy risk in children (2·66, 1·10–6·49). The increased IRR values calculated for children with familial incomes lower than the minimum wage were not significant (table 3).

Proximal factors associated with an increased leprosy risk in the total population and in children included the lack of a public network for sewage disposal and living in an accommodation built of less durable materials than brick and cement (table 2; table 3). IRR estimates for housing material other than brick and cement were 1·34 (95% CI 1·29–1·40) in the full cohort and 1·26 (1·11–1·43) for children younger than 15 years. The leprosy IRR for absence of public sewage disposal was 1·35 (1·30–1·40) in the total cohort and 1·55 (1·37–1·75) for children. Absence of a formal lighting source in the home was also associated with increased risk of leprosy in children (1·19, 1·05–1·35). There was no evidence of an association of household water supply, electricity in family home, waste collection system, or housing density with leprosy risk in the total cohort. In children, use of a non-public waste collection system was inversely associated with leprosy risk (0·79, 0·68–0·93), whereas increased housing density was associated with an increase in leprosy risk (1·21, 1·01–1·44, for more than one person per room *vs* less than one person per two rooms).

Increasing age was associated with an increased leprosy risk in the total cohort and in children. The IRR of leprosy in the proximal model was 1·36 (95% CI 1·35–1·36) per 10-year increase in age in the full cohort, and 3·33 (2·92–3·80) in children (10–15 years *vs* 0–5 years). Male sex was also associated with an increased leprosy incidence in the full cohort (1·22, 1·19–1·26) in the proximal model, but not in children.

In the analysis stratified by leprosy subtypes, results were similar between paucibacillary (7310) and multi-bacillary (11 205) cases for most socioeconomic determinants (appendix pp 2–3). Age, region, location of family home, race or ethnicity, education, employment and income were associated with risk of both paucibacillary and multibacillary leprosy. Similar trends were apparent for both disease subtypes across all factors with the exception of sex, which showed opposing directions of association between paucibacillary (male *vs* female IRR 0·73, 95% CI 0·70–0·77) and multibacillary subtypes (1·70, 1·63–1·76; appendix pp 2–3).

Stratified analyses by geographical region showed little evidence of differences between regions for most socioeconomic determinants of leprosy risk with the exception of education. The inverse association of education with leprosy risk, which was evident in all regions, appeared to be strongest in the south and south-east regions (appendix pp 4–5).

Sensitivity analyses adjusting for year of entry into the cohort and restricted follow-up time to 2 years showed consistent results with the primary analysis (appendix pp 6–9).

## Discussion

In this study, we have investigated the association of socioeconomic and demographic factors with leprosy incidence in the 100 Million Brazilian Cohort. With 33 877 938 individuals, including 23 911 leprosy cases, to our knowledge, this is the largest study to investigate the social determinants of leprosy.

Study results provided strong evidence of an association of poverty indicators with leprosy incidence. Factors that showed a consistent direction of effect across analyses included age, region of residence, location of family home, race or ethnicity, education, income, and indicators of living conditions (ie, housing material and sewage disposal system). The direction of association for these factors in both primary and subgroup analyses, found evidence that the most deprived groups in Brazil are at the greatest risk of leprosy detection. Individuals residing in regions with the most widespread poverty in the country (central-west, north, and northeast regions) had leprosy risk five-to-eight times greater than other individuals.^20^ Similarly, being of a self-reported “preto” (ie, black) or “pardo” (ie, mixed race) ethnicity—which is associated with higher deprivation levels in Brazil—was linked to an up to 40% increase in disease risk.^21^ However, Indigenous ethnicity, which is also associated with increased levels of poverty in Brazil, was associated with decreased risks of leprosy in our study.^21^ The lower detection rates in Indigenous individuals than in other ethnic groups could reflect lower prevalence of disease because of their living in isolated communities and perhaps an under-diagnosis of leprosy cases because of disparities in health-care access.^22^ The difference in risk across ethnic groups increased in individuals younger than 15 years, with results showing a 92% increase in leprosy incidence in children who identified as “preto” (ie, black) compared with those who identified as “branco” (ie, white). Furthermore, direct indicators of deprivation, including no family income, lower level of education, and factors reflecting unfavourable living conditions, were associated with a leprosy incidence up to two-times higher. A gradient effect was evident, showing an increasing risk of leprosy with decreasing income and education level.

The association of unfavourable socioeconomic conditions with increased leprosy risk has been reported in previous studies and was recently summarised in a systematic review by Pescarini and colleagues.^7^ These authors reported a number of poverty indicators associated with an increased risk of leprosy in high-burden countries, including food shortages, increased prevalence of illiteracy, and decreased income. Among studies included in the review, increased prevalence of illiteracy was associated with an up to two-times greater risk of leprosy, whereas decreased income was associated with an increased leprosy risk in one out of four studies.

Additional socioeconomic factors that reflect increased contact exposure and increased levels of deprivation, such as urbanisation and household crowding, have also been associated with an increased risk of leprosy detection.^7^, ^23^ Although we found similar evidence that individuals living in urban areas were at a greater risk of leprosy detection than individuals living in rural areas, we did not find evidence of an association of household density with leprosy risk in the full cohort. It is, however, noteworthy that in subgroup analyses increased household density (more than one resident per two rooms) was associated with an increased leprosy risk in children, a group indicative of active transmission. Most countries reporting sex-specific leprosy incidence report more detected cases in men than in women,^24^, ^25^ which is in line with our study findings. This sex-associated difference in leprosy detection has been suggested to reflect differences between men and women in exposure behaviours and health-care access.^25^, ^26^, ^27^

Only one individual-level study has previously assessed the association of unemployment with leprosy risk and found no evidence of an association.^28^ In our study, we found that unemployment was associated with a decreased leprosy risk, despite the crude analysis indicating increased leprosy incidence in unemployed individuals. However, after accounting for changes to the CadÚnico registration questionnaire, this protective effect appeared to be driven by the 18–30-year age subpopulation. This inverse association might be due to different behavioural patterns between employed and unemployed individuals in this age group. However, we were unable to investigate this association further because of the unavailability of detailed information in the registry data.

There are several strengths to this study. As leprosy is a rare disease with a long incubation period, accrual of a sufficient number of cases represents an important limitation to longitudinal studies of the disease. The large size of our study population, with a follow-up duration of up to 8 years, has provided us with unprecedented power to study key socioeconomic determinants of leprosy. Furthermore, we have performed the largest individual-level prospective investigation of poverty and leprosy, providing a more robust estimation of the effect of deprivation on leprosy than has previously been possible. The detailed level of data on income and education available in this study has also enabled us to assess the gradient impact of these factors on leprosy risk.

However, our study has limitations. As the data used was routinely collected for non-research purposes, there are limitations to the detail and completeness of the information collected. For instance, other socioeconomic determinants—such as household cleanliness, nutrition, and food shortages—that have been associated with leprosy risk in previous studies were not available in this study. The level of information available has also restricted our ability to investigate the specific mechanisms through which socioeconomic factors might influence leprosy infection and transmission (eg, as related to the unexpected inverse association of unemployment with leprosy incidence in individuals aged 18–30 years). Additionally, although we adjusted for region of residence, data for relocation and migration of cohort members were not available. This could have led to attenuated estimates of leprosy risk, if relocation was associated with a change in socioeconomic status.

Missing data are a common problem for routinely collected records. Overall, we excluded 29% of the population because of missing information, particularly related to education and employment. As the leprosy incidence in the complete-case analysis was similar to that of the total population (18·7 per 100 000 for the complete-case population *vs* 17·1 per 100 000 for the total population), the exclusion of participants with missing data is unlikely to have strongly biased our findings. Of note, however, leprosy incidence was lower in individuals with missing data for education (13·1 per 100 000) than in those for whom these data were available (17·8 per 100 000), and in individuals with missing data for employment (11·3 per 100 000) than in those for whom these data were available (18·2 per 100 000); these differences might have influenced our estimates for these factors.

As information on leprosy cases diagnosed before 2007 was not included, individuals diagnosed before 2007 who have not completed treatment might be misclassified in our population. However, given the rarity of the disease, this is unlikely to have had any substantial effect on study results. Finally, as the population used in this study consists of potential beneficiaries of social protection programmes, who represent the poorest portion of the Brazilian population, the findings of this study might not be applicable to the general Brazilian population. Indeed, the difference in risk between our study population and individuals in the higher socioeconomic strata of the Brazilian population is likely to be even greater.

The findings of this study show that the most deprived segments of the Brazilian population are at greatest risk of leprosy. The existence of a gradient in disease risk with increasing poverty within the poorest portion of the Brazilian population is a strong argument for the important contribution of deprivation to leprosy risk. This unequal distribution of risk places an even greater burden on already socioeconomically disadvantaged groups, reinforcing existing social and health inequalities.

Our results have important implications for the strategy of disease control in Brazil and abroad. Early detection and prevention in high-risk communities is vital for interrupting leprosy transmission in children and reducing the prevalence of stigmatising leprosy-related disabilities. Strategies aiming to increase leprosy diagnosis and improve access to health care in the poorest populations in leprosy-endemic regions might have important benefits for improving disease control and achieving WHO goals. Our results support the existing evidence, which suggests that along with early diagnosis and treatment of leprosy cases, social development is a key strategy for reducing the prevalence of leprosy worldwide.^29^ Poverty alleviating interventions, such as the Brazilian cash transfer programme Bolsa Familia, could substantially contribute towards achieving the goal of leprosy elimination.^30^, ^31^
