# Supplementary material for: Socioeconomic determinants of leprosy new case detection in the 100 Million Brazilian Cohort: a population-based linkage study
Source: Lancet Glob Health. 2019 Jul 19;7(9):e1226–36. doi: 10.1016/S2214-109X(19)30260-8 (PMC6688099; doi:10.1016/S2214-109X(19)30260-8)
Supplement: Supplementary appendix [file mmc1.pdf]

THE LANCET
Global Health

Supplementary appendix

This appendix formed part of the original submission and has been peer reviewed. We post it as supplied by the authors.

Supplement to: Nery JS, Ramond A, Pescarini JM. Socioeconomic determinants of leprosy new case detection in the 100 Million Brazilian Cohort: a population-based linkage study. *Lancet Glob Health* 2019; published online July 19. http://dx.doi.org/10.1016/S2214-109X(19)30260-8.

Supplementary material

Supplementary Table 1 Association of employment with leprosy in participants >18y, stratified by time period of registration.

Age groups (y)	Currently employed	Unemployed student		Unemployed	
		IRR (95%CI)	P-value	IRR (95%CI)	P-value
**CadÚnico registration: 2007-2009**					
18-30	Ref	0.86 (0.80, 0.92)	<0.001	0.79 (0.71, 0.89)	<0.001
30-40	Ref	0.90 (0.82, 0.98)	0.01	1.04 (0.91, 1.19)	0.57
40-50	Ref	0.91 (0.82, 1.01)	0.07	1.18 (1.03, 1.36)	0.02
50-60	Ref	0.78 (0.70, 0.88)	<0.001	1.08 (0.95, 1.23)	0.23
>60	Ref	0.69 (0.61, 0.78)	<0.001	0.78 (0.63, 0.82)	<0.001
**CadÚnico registration: 2011-2014**					
18-30	Ref	0.86 (0.80, 0.92)	<0.001	0.74 (0.71, 0.89)	<0.001
30-40	Ref	0.89 (0.37, 2.15)	0.79	1.06 (0.90, 1.26)	0.47
40-50	Ref	0.33 (0.05, 2.37)	0.27	1.06 (0.88, 1.27)	0.53
50-60	Ref	1.02 (0.33, 3.19)	0.97	0.88 (0.75, 1.03)	0.12
>60	Ref	1.52 (0.48, 4.81)	0.48	1.07 (0.85, 1.37)	0.53

IRR: incidence rate ratio for leprosy new case detection

IRRs were obtained using generalized linear Poisson models with clustered standard errors to account for clustering by family. As the questionnaire used to record employment changed in 2010 , the analysis is stratified from 2007-2009 and 2011-2014.

Supplementary Figure 1 Forest plot of multivariate association of distal and intermediate socio-demographic determinants with paucibacillary and multibacillary leprosy

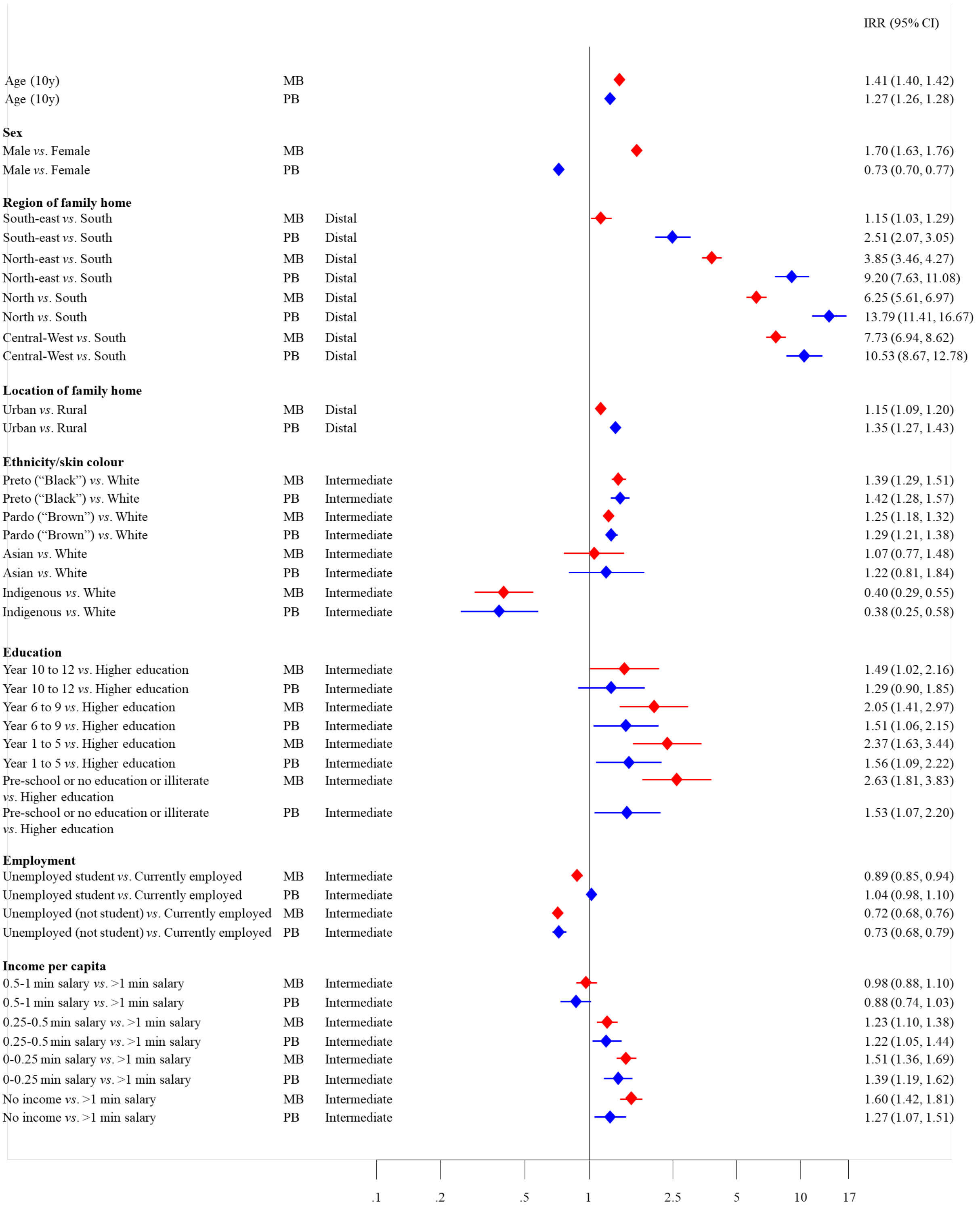

IRR: incidence rate ratio for leprosy new case detection, MB: multibacillary, PB: paucibacillary
IRRs were obtained using generalized linear Poisson models with clustered standard errors to account for clustering by family. A complete case analysis approach was adopted excluding individuals with missing data in any of the three models from all models.

Supplementary Figure 2 Forest plot of multivariate association of proximal socio-demographic determinants with paucibacillary and multibacillary leprosy

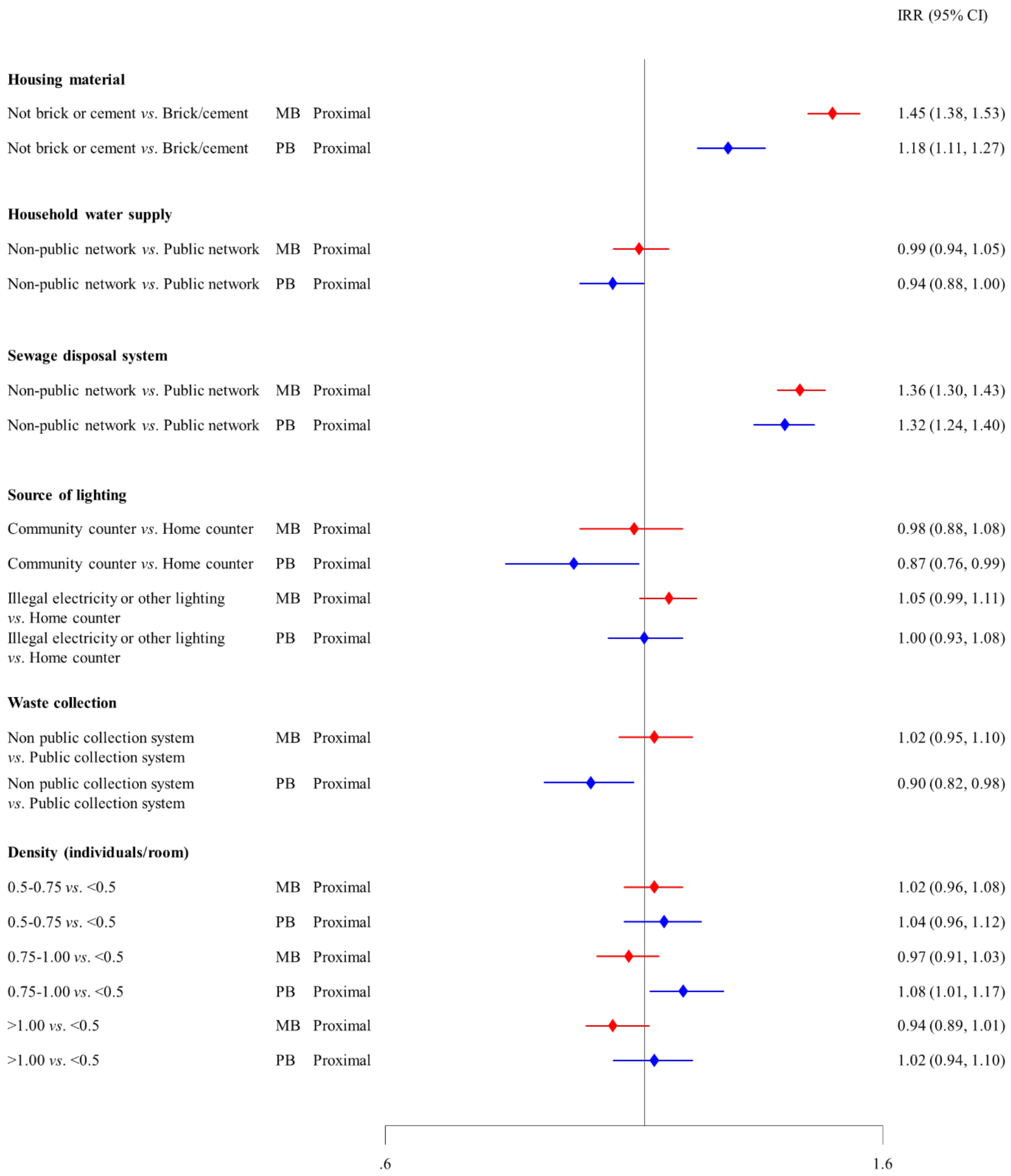

IRR: incidence rate ratio for leprosy new case detection, MB: multibacillary, PB: paucibacillary
IRRs were obtained using generalized linear Poisson models with clustered standard errors to account for clustering by family. A complete case analysis approach was adopted excluding individuals with missing data in any of the three models from all models.

Supplementary Figure 3 Forest-plot of multivariate association of distal and intermediate socio-demographic determinants with leprosy by region of residence

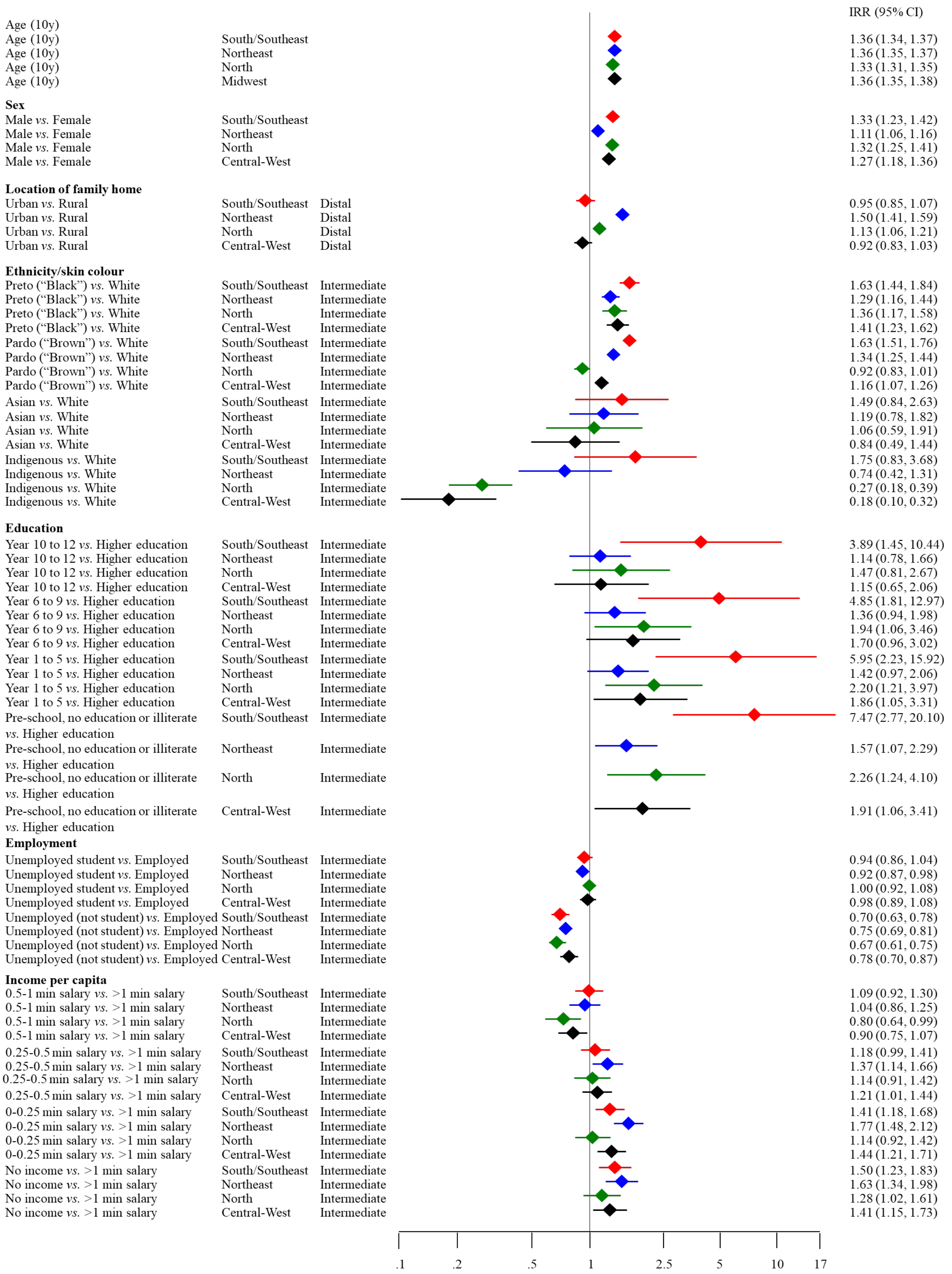

IRR: incidence rate ratio for leprosy new case detection. IRRs were obtained using generalized linear Poisson models with clustered standard errors to account for clustering by family. A complete case analysis approach was adopted excluding individuals with missing data in any of the three models from all models.

Supplementary Figure 4 Forest-plot of multivariate association of proximal socio-demographic determinants with leprosy by region of residence

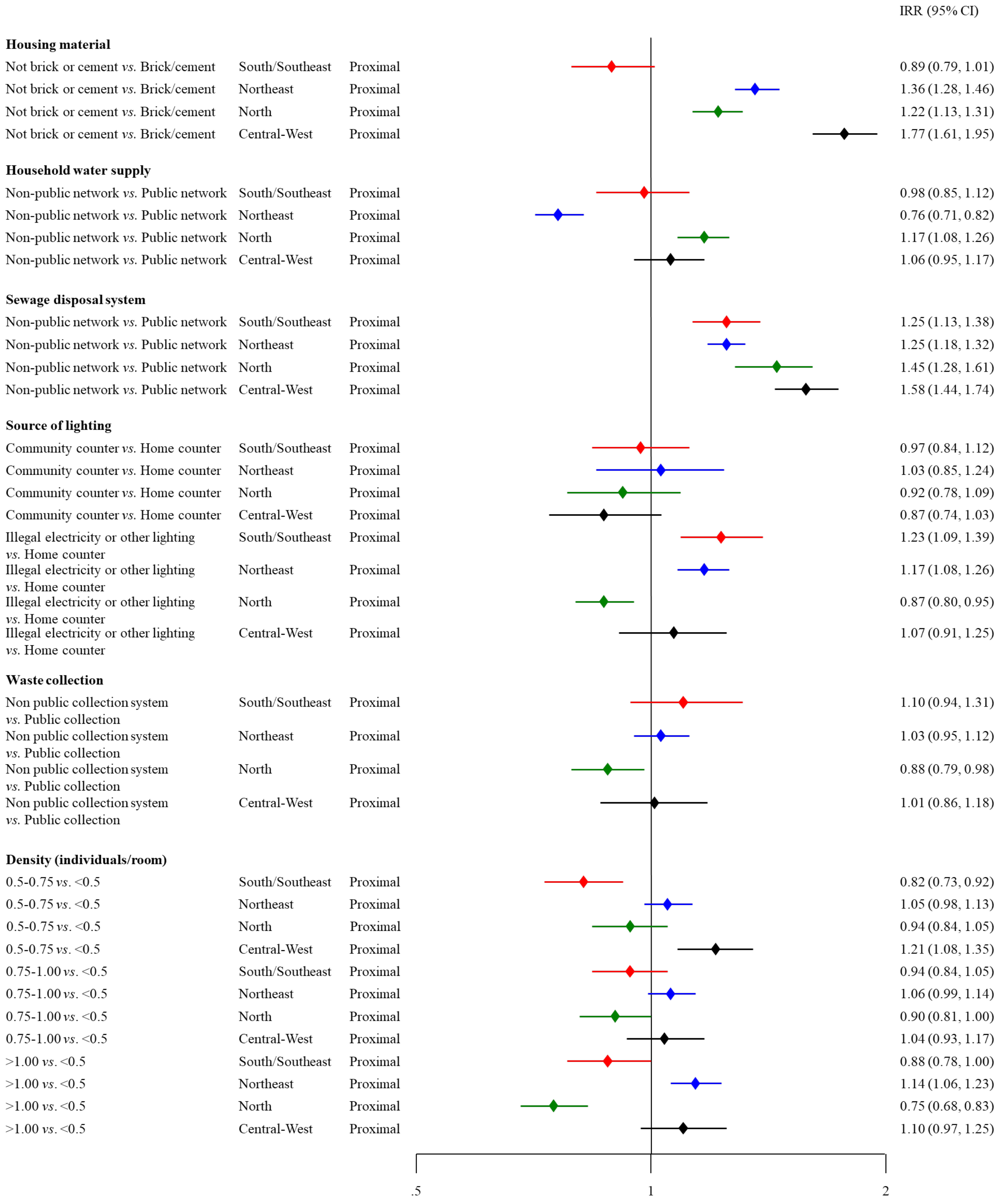

IRR: incidence rate ratio for leprosy new case detection. IRRs were obtained using generalized linear Poisson models with clustered standard errors to account for clustering by family. A complete case analysis approach was adopted excluding individuals with missing data in any of the three models from all models.

Supplementary Table 2 Multivariate hierarchical association of socioeconomic factors with leprosy incidence adjusting for year of entry into the 100 Million Brazilian cohort

	Model 1		Model 2 *		Model 3 **	
**Variable**	**IRR**	**P-value**	**IRR**	**P-value**	**IRR**	**P-value**
**Age (per 10y)**	1.32 (1.32, 1.33)	<0.001	1.35 (1.34, 1.36)	<0.001	1.36 (1.35, 1.36)	<0.001
**Sex**						
Female	Ref					
Male	1.25 (1.21, 1.28)	<0.001	1.22 (1.19, 1.26)	<0.001	1.22 (1.19, 1.26)	<0.001
**Year at baseline**						
2007	Ref		Ref		Ref	
2008	0.82 (0.79, 0.86)	<0.001	0.85 (0.82, 0.89)	<0.001	0.87 (0.83, 0.90)	<0.001
2009	0.81 (0.78, 0.85)	<0.001	0.84 (0.80, 0.88)	<0.001	0.86 (0.82, 0.91)	<0.001
2010	0.77 (0.72, 0.82)	<0.001	0.80 (0.75, 0.86)	<0.001	0.83 (0.77, 0.88)	<0.001
2011	0.62 (0.57, 0.66)	<0.001	0.73 (0.68, 0.79)	<0.001	0.75 (0.70, 0.81)	<0.001
2012	0.55 (0.52, 0.59)	<0.001	0.67 (0.59, 0.72)	<0.001	0.69 (0.65, 0.74)	<0.001
2013	0.54 (0.49, 0.60)	<0.001	0.65 (0.59, 0.72)	<0.001	0.67 (0.61, 0.75)	<0.001
2014	0.60 (0.53, 0.68)	<0.001	0.74 (0.64, 0.85)	<0.001	0.76 (0.67, 0.88)	<0.001
**Distal variables**						
**Region of family home**						
South	Ref					
South-east	1.43 (1.30, 1.58)	<0.001				
North-east	4.71 (4.30, 5.16)	<0.001				
North	7.47 (6.80, 8.20)	<0.001				
Central-West	8.30 (7.55, 9.13)	<0.001				
**Location of family home**						
Rural	Ref					
Urban	1.28 (1.23, 1.33)	<0.001				
**Intermediate variables**						
**Ethnicity/skin colour**						
White			Ref			
Preto (“Black”)			1.40 (1.31, 1.49)	<0.001		
Pardo (“Brown”)			1.26 (1.21, 1.32)	<0.001		
Asian			1.18 (0.91, 1.52)	0.21		
Indigenous			0.39 (0.30, 0.51)	<0.001		
**Highest level of education*** **						
Higher education			Ref			
Year 10 to 12			1.48 (1.13, 1.94)	0.004		
Year 6 to 9			1.79 (1.37, 2.34)	<0.001		
Year 1 to 5			1.96 (1.50, 2.56)	<0.001		
Pre-school or no education or illiterate			1.94 (1.48, 2.54)	<0.001		
**Employment*** **						
Currently employed			Ref			
Unemployed student			0.90 (0.87, 0.94)	<0.001		
Unemployed (not student)			0.81 (0.77, 0.85)	<0.001		
**Income per capita**						
>1 min salary			Ref			
0.5-1 min salary			0.96 (0.88, 1.06)	0.45		
0.25-0.5 min salary			1.20 (1.09, 1.32)	<0.001		
0-0.25 min salary			1.38 (1.26, 1.52)	<0.001		

No income	1.41 (1.28, 1.56)	<0.001		
**Proximal variables**				
**Housing material**				
Brick or cement			Ref	
Taipa/Wood/Other			1.32 (1.27, 1.38)	<0.001
**Household water supply**				
Public network			Ref	
Well/Natural source/Cistern/Other			0.97 (0.93, 1.01)	0.12
**Sewage disposal system**				
Public network			Ref	
Septic tank/Ditch/Other			1.34 (1.29, 1.39)	<0.001
**Electricity in family home**				
Home counter			Ref	
Community counter			0.96 (0.88, 1.04)	0.31
Illegal electricity/Gas lighting/Candlelight/Other			1.03 (0.98, 1.08)	0.18
**Waste collection system**				
Public collection system			Ref	
Burned/Buried/Outdoor disposal/Other			0.97 (0.91, 1.02)	0.17
**Density (individuals/room)**				
<=0.5			Ref	
0.5-0.75			1.01 (0.96, 1.06)	0.66
0.75-1.00			0.99 (0.94, 1.04)	0.69
>1.00			0.94 (0.89, 0.98)	0.008

IRR: incidence rate ratio for leprosy new case detection

IRRs were obtained using generalized linear Poisson models with clustered standard errors to account for clustering by family. A complete case analysis approach was adopted excluding individuals with missing data in any of the three models from all models.

*Model 2: covariates in Model 2 are adjusted for covariates from Model 1 with a p-value<0.1, i.e. Model 2 is adjusted for region and location of family home

**Model 3: covariates from Model 3 are adjusted for covariates from Model 1 and Model 2 with a p-value<0.1, i.e. Model 3 is adjusted for region, location of family home, ethnicity, education, employment, and income

***Information on education and employment are reported at the individual level for adult individuals (>18y) and for the oldest member of the family for individuals aged under 18y

Supplementary Table 3 Multivariate hierarchical association of socioeconomic factors with leprosy incidence restricted to 2 years of follow-up (N=23,670,871)

	**Model 1**		**Model 2 ***		**Model 3 ****	
**Variable**	**IRR**	**P-value**	**IRR**	**P-value**	**IRR**	**P-value**
**Age (per 10y)**	1.33 (1.32, 1.34)	<0.001	1.38 (1.37, 1.39)	<0.001	1.38 (1.37, 1.39)	<0.001
**Sex**						
Female	Ref					
Male	1.32 (1.26, 1.38)	<0.001	1.29 (1.24, 1.36)	<0.001	1.29 (1.23, 1.35)	<0.001
**Distal variables**						
**Region of family home**						
South	Ref					
South-east	1.55 (1.35, 1.78)	<0.001				
North-east	5.14 (4.51, 5.87)	<0.001				
North	8.67 (7.58, 9.94)	<0.001				
Central-West	8.30 (7.23, 9.51)	<0.001				
**Location of family home**						
Rural	Ref					
Urban	1.23 (1.16, 1.31)	<0.001				
**Intermediate variables**						
**Ethnicity/skin colour**						
White			Ref			
Preto ("Black")			1.46 (1.33, 1.61)	<0.001		
Pardo ("Brown")			1.28 (1.20, 1.36)	<0.001		
Asian			0.95 (0.64, 1.42)	0.81		
Indigenous			0.49 (0.33, 0.73)	<0.001		
**Highest level of education*****						
Higher education			Ref			
Year 10 to 12			1.45 (0.93, 2.25)	0.102		
Year 6 to 9			1.92 (1.24, 2.98)	0.003		
Year 1 to 5			2.09 (1.35, 3.24)	0.001		
Pre-school or no education or illiterate			2.22 (1.42, 3.45)	<0.001		
**Employment*****						
Currently employed			Ref			
Unemployed student			1.06 (0.99, 1.12)	0.09		
Unemployed (not student)			0.73 (0.68, 0.78)	<0.001		
**Income per capita**						
>1 min salary			Ref			
0.5-1 min salary			0.88 (0.78, 0.99)	0.04		
0.25-0.5 min salary			1.12 (0.99, 1.27)	0.08		
0-0.25 min salary			1.39 (1.23, 1.57)	<0.001		
No income			1.49 (1.29, 1.71)	<0.001		
**Proximal variables**						
**Housing material**						
Brick or cement					Ref	
Taipa/Wood/Other					1.32 (1.24, 1.41)	<0.001
**Household water supply**						
Public network					Ref	
Well/Natural source/Cistern/Other					1.00 (0.93, 1.07)	0.97
**Sewage disposal system**						
Public network					Ref	

Septic tank/Ditch/Other	1.34 (1.26, 1.42)	<0.001
**Electricity in family home**		
Home counter	Ref	
Community counter	1.08 (0.97, 1.22)	0.31
Illegal electricity/Gas lighting/Candlelight/Other	1.08 (1.00, 1.16)	0.06
**Waste collection system**		
Public collection system	Ref	
Burned/Buried/Outdoor disposal/Other	1.00 (0.91, 1.09)	0.93
**Density (individuals/room)**		
<=0.5	Ref	
0.5-0.75	0.96 (0.89, 1.03)	0.23
0.75-1.00	1.00 (0.93, 1.07)	0.92
>1.00	0.87 (0.80, 0.94)	0.001

IRR: incidence rate ratio for leprosy new case detection

IRRs were obtained using generalized linear Poisson models with clustered standard errors to account for clustering by family. A complete case analysis approach was adopted excluding individuals with missing data in any of the three models from all models.

*Model 2: covariates in Model 2 are adjusted for covariates from Model 1 with a p-value<0.1, i.e. Model 2 is adjusted for region and location of family home

**Model 3: covariates from Model 3 are adjusted for covariates from Model 1 and Model 2 with a p-value<0.1, i.e. Model 3 is adjusted for region, location of family home, ethnicity, education, employment, and income

***Information on education and employment are reported at the individual level for adult individuals (>18y) and for the oldest member of the family for individuals aged under 18y
